# Continuous Interstitial Glucose Monitoring through a Long-Term Subcutaneous Implantable Sensor during Pregnancy in Type 1 Diabetes: A Two-Patient Case Report

**DOI:** 10.2174/0118715303393466250807050121

**Published:** 2025-08-18

**Authors:** Betella Nazarena, Resi Veronica, Gaglio Alessia, Ciafardini Antea, Mazzoleni Federica, Vena Walter, Orsi Emanuela, Bossi Antonio Carlo

**Affiliations:** 1 Endocrinology and Diabetology Unit, Humanitas Gavazzeni Institute, via M. Gavazzeni 21, 24100, Bergamo, Italy;; 2 Endocrinology and Metabolic Diseases Unit, Fondazione IRCCS Ca' Granda - Ospedale Maggiore Policlinico, Milan, Italy;; 3 Endocrinology, Diabetology and Andrology Unit, Humanitas Clinical and Research Center IRCCS, Rozzano (MI), Italy

**Keywords:** Type 1 diabetes, glucose monitoring, pregnancy, gestational outcomes, insulin dose, subcutaneous implantable sensor

## Abstract

**Introduction:**

Optimal glycaemic control is crucial during pregnancy in women with type 1 diabetes mellitus (T1D). Current guidelines, based on positive data from the CONCEPTT (Continuous Glucose Monitoring in Pregnant Women With Type 1 Diabetes Trial), on maternal glycaemic control and fetal outcomes, recommend offering real-time Continuous Glucose Monitoring (rt-CGM) as a standard method in all pregnancies of women with type 1 diabetes (T1D).

**Case Presentation:**

In these two clinical cases, we describe for the first time the gestational outcomes in two patients with T1D who chose to maintain a long-term implantable subcutaneous sensor (Eversense XL^®^) as a CGM method during their pregnancies. The first case concerns a 33-year-old young woman with a 25-year history of T1D on Continuous Subcutaneous Insulin Infusion (CSII), who faced her first pregnancy with a suboptimal preconception glycaemic control; the second case describes the second pregnancy of a 37-year-old patient with a more recently diagnosed T1D, on multiple daily injection (MDI) therapy who achieved adequate glycaemic compensation before planned conception.

**Conclusion:**

The subcutaneous sensor replacement was carried out at the beginning and end of the 2^nd^ trimester, respectively, without any complications, allowing optimal monitoring and adjustment of insulin dose and achieving optimal glucose targets throughout the pregnancy until delivery.

## 1 INTRODUCTION

The optimization of glycaemic control is of primary importance in the pregnancy of women affected by type 1 diabetes mellitus (T1D), where the risk of adverse maternal-fetal outcomes correlates with disease control [1]. Whilst 95% of mothers have successful liveborn babies, obstetric and neonatal complications related to maternal hyperglycaemia remain ubiquitous, affecting one in two pregnancies complicated by T1D [2]. These include preterm births (delivery before 37 weeks’ gestation), large for gestational age (LGA) birthweight (>90th percentile), and neonatal care unit admissions. Recent UK data suggest improvements in periconception glycaemia among women with type 1 diabetes (T1D), most likely attributed to increased diabetes technology use during 2021-22 [3]. Continuous glucose monitoring (CGM), allowing for real-time adjustments of insulin therapy or based on the retrospective analysis of glycaemic trends, was shown to be superior to other monitoring systems (capillary and interstitial 'on demand') in improving periconception glucose levels, with slightly more users achieving target glycated haemoglobin (HbA1c) in early pregnancy, improved maternal glycaemic control, with significantly more users achieving target HbA1c after 24 weeks’ gestation, and better obstetric and neonatal outcomes [4]. Based on these data, the UK National Institute for Health and Care Excellence (NICE) recommends that all pregnant women with T1D be offered CGM [5]. However, there is currently no literature evidence regarding the glycaemic monitoring and gestational outcomes of patients with T1D undergoing CGM through a long-term (up to 180-day long) subcutaneous implantable sensor (Eversense XL^®^) [6].

## 2 CASE PRESENTATION

### 2.1 Case 1

A 33-year-old woman with T1D since 1997, treated with Continuous Subcutaneous Insulin Infusion (CSII, Omnipod DASH^®^) and continuous glucose monitoring (CGM) through a long-term subcutaneous sensor implanted in the pre-conception period, presented with fair (but not optimal) pre-pregnancy glycaemic control at her last visit on August 23rd, 2022. She returned for removal and re-implantation of the sensor on November 11th, 2022, informing us that she was pregnant, at 13 weeks’ gestation. She was offered a validated CSII + CGM system adapted for pregnancy; however, she preferred to continue with her current treatment and monitoring system, with which she felt more confident. During pregnancy, the patient replaced the implantable system without any adverse events.

We noted poor adherence to dietary-nutritional advice, resulting in a total weight gain of +15 kg (recommended < 11 kg for normal-weight individuals like our patient, whose pre-pregnancy BMI was 22.5 kg/m^2^). We modified insulin infusion rates accordingly (total daily basal insulin was 15.6 U/day in early pregnancy and 24.1 U/day before delivery) and the prandial insulin-to-carbohydrate ratios (1:20 at breakfast, 1:15 at lunch and 1:18 at dinner corresponding to a mean bolus dose of 2/5/5 U in early pregnancy; 1:12 at breakfast, 1:12 at lunch and 1:16 at dinner corresponding to an average bolus dose of 3.5/5.5/5.5 U at end of pregnancy), consistent with a total daily insulin dose of 27.6 U (0.51 U/kg/day) and 38.6 U (0.56 U/kg/day), respectively, in early pregnancy and before delivery. Despite a persistent tendency to underestimate the dose of the evening insulin bolus due to fear of nocturnal hypoglycaemia, leading to consistently high post-dinner blood glucose levels, the composite outcome of HbA1c and time spent in euglycaemia/hyperglycaemia was fully achieved in the second and third trimesters, while an appropriate time spent in hypoglycaemia was achieved only in the third trimester. Fetal growth parameters remained consistently within normal limits (abdominal circumference <50th percentile, regular amniotic fluid) throughout the pregnancy (Table **1**). Delivery was carried out on May 1st, 2023, at 38+4 gestational weeks through a scheduled induction of labour, resulting in the birth of a baby girl (weighing 2,950 grams).

### 2.2 Case 2

A 37-year-old woman, diagnosed with T1D in 2019 after a first pregnancy in 2013 complicated by gestational diabetes mellitus (delivery at 36 weeks of gestation, neonatal weight of 3160 grams, neonatal hypoglycaemia and jaundice), planned a second pregnancy after achieving adequate glycaemic control, as indicated by a May 2023 HbA1c value of 6.4%. She refused to switch to CSII treatment due to personal preference and continued multiple daily injections (MDI), consisting of a morning administration of insulin glargine U300 and three daily injections of insulin aspart based on carbohydrate counting, monitored with a CGM system *via* a 6-month implantable subcutaneous sensor that she had been using regularly since 2021. She was informed about the approved and conventionally used tools for CGM in pregnancy; however, she decided not to change the type of sensor to which she was accustomed. Consequently, the subcutaneous sensor was successfully removed and reimplanted on November 16th, 2023, at 26 weeks’ gestation. Apart from levothyroxine replacement therapy for autoimmune hypothyroidism (TSH 10.67 mU/L) started at the 10^th^ week and metoprolol 25 mg indicated from the 32^nd^ gestational week for symptomatic atrial tachycardia, the pregnancy proceeded without complications, achieving good control for both conditions (TSH = 1.28 mU/L in November 2023, HR = 73 bpm in January 2024). Basal insulin dosage increased from 0.20 to 0.35 U/kg/day (from 11 to 24 units; while insulin aspart doses were calculated at each meal according to carb counting based on an insulin/carbohydrate ratio of 1:3 at breakfast and 1:4 at lunch and dinner), consistent with an overall weight gain of +14 kg (recommended < 11 kg for normal-weight individuals like our patient, whose pre-pregnancy BMI was 21.3 kg/m^2^). Despite a slightly excessive amount of time spent in hypoglycaemia in the second-third trimester, the composite outcome of HbA1c and time spent in euglycaemia/hyperglycaemia was fully achieved during this period, which could not be achieved in the first trimester only due to a slightly longer-than-expected time spent in hyperglycaemia, but with an excellent goal of time spent in hypoglycaemia. Fetal growth parameters (Abdominal Circumference, Estimated Fetal Weight, and Amniotic Fluid Index) remained consistently between the 30^th^ and 40^th^ percentiles throughout pregnancy (Table **2**). Delivery was carried out on February 23^rd^, 2024 at 39+3 gestational weeks through a scheduled induction of labour, resulting in the birth of a baby girl (weighing 2.900 grams).

## 3 DISCUSSION

Analysis of CGM profiles suggests that optimizing maternal glucose levels by 10 to 12 weeks’ gestation is key to preventing stagnation and/or deterioration of antenatal glycaemia in mid-gestation, which is strongly associated with fetal growth acceleration and LGA birthweight [7, 8]. In addition to appropriate HbA1c levels (<6.5% pre-conception and in the 1st trimester; <6.0% in the 2nd and 3rd trimesters), the glycaemic targets to be achieved included increasing the time spent in euglycaemia (Time In Range, TIR: 63–140 mg/dL, >70%) compared to time spent in hyperglycaemia (Time Above Range, TAR: >140 mg/dL, <25%) or hypoglycaemia (Time Below Range, TBR: <63 mg/dL, <4%; <54 mg/dL, <1%), while reducing glycaemic variability [9, 10].

Unfortunately, HbA1c levels and sensor glycaemic parameters were not adequate preconceptionally in the first patient's unplanned pregnancy, and although the TIR reached the target in both patients during early pregnancy (I^st^ trimester), the HbA1c value remained above the recommended limit in the first patient, and the TAR level exceeded the recommended upper limit in the second patient. Achieving the stringent pregnancy glucose targets of HbA1c <43 mmol/mol (6.1%) and TIR ≥ 70% was accomplished by only ~33% of CGM users, regardless of insulin delivery method (pump or multiple daily injections) [9]. Current evidence, based on results from the Automated insulin Delivery Amongst Pregnant women with T1D (AiDAPT) study, suggested that the adaptive hybrid closed loop (HCL) system (CamAPS^®^ FX) was the most effective means of rapidly optimizing maternal glycaemia in early pregnancy, as 61 pump-naive women randomized to the HCL system achieved greater improvements in maternal outcomes (including +5% I-trimester TIR within days of starting HCL system use, +10.5% TIR from 16 weeks’ gestation through delivery, -10% TAR, less time in overnight hypoglycaemia, -3.7 kg unexpected gestational weight gain) compared to 63 pump-naive women randomized to CGM (Dexcom G6^®^) along with their usual insulin therapy [11]. Therefore, the NICE clinical guidelines recommend that an HCL system should ideally be offered before pregnancy to all ‘women, trans men, and non-binary people with type 1 diabetes who are planning to become pregnant or at least as soon as possible after confirmation of pregnancy’ in order to reduce obstetric and neonatal complications in unplanned pregnancies [12]. However, the AiDAPT results are applicable only to the CamAPS^®^ FX system, which has an adaptive algorithm (adapting over 24 h, after meals and day-to-day), lower glucose targets (97 mg/dl [5.4 mmol/L] and 93 mg/dl [51 mmol/L] in the II and III trimesters, respectively) and is the most extensively studied system in T1D pregnancy [13, 14]. Moreover, an increase in TAR by +5%, alongside a decrease in TIR by -5% in the 2nd and 3rd trimesters, is known to be associated with a major risk of large for gestational age (LGA) newborns, subsequent hypoglycaemia, and neonatal intensive care unit admissions [15]. Although our patients chose only CGM, refusing the HCL system, from the second trimester onwards, throughout pregnancy and in the immediate postpartum period, all parameters, except TBR and overall maternal weight gain (just as observed in the AiDAPT study), were satisfactory in both patients, without causing subsequent neonatal adverse events. Eversense^®^ requires twice-daily calibration (every 12 hours, in the morning and in the evening). Additionally, patients were advised to verify the accuracy of the implantable subcutaneous sensor measurements using their capillary glucose meter whenever the glucose value indicated by the sensor was considered inaccurate or inconsistent, especially if this occurred in particular circumstances (*e.g.*, prior to an insulin dose adjustment). The use of a non-validated tool for pregnancy did not lead to an intensification in the frequency of visits, which were almost always fortnightly, given the possibility of consulting daily glucose values *via* a Data Management System (Pro Eversense DMS) for remote evaluation. Both patients were well informed that Eversense^®^ was not an approved method for glucose monitoring during pregnancy due to the lack of data in this population, but they still felt more confident continuing to use a method they had been familiar with for the past few years and had learned to manage accordingly, never changing their minds about it throughout the whole pregnancy period. The significant increase in sensor longevity is one of the most notable advantages of this abiotic fluorescence CGM system over commercially available transcutaneous CGM devices, which use enzymes with limited lifespans due to thermal degradation, limiting their *in vivo* life to up to 15 days [16]. In contrast, the subcutaneously inserted fluorescent sensor detects glucose using a non-enzymatic methodology, which does not protrude from the skin and is not subject to surface phenomena that can alter the electrode characteristics and compromise its performance [16]. Eversense^®^ is fully inserted into the interstitial tissue, allowing the body to heal the insertion wound and resolve the acute inflammatory response. The MARD of 11.67% is comparable to or higher than that reported with commercial CGMs with much shorter durations [16].

## CONCLUSION

This observation presents our preliminary experience with the 6-month subcutaneous implantable sensor, including its explantation and reimplantation, during a planned and an unplanned pregnancy in two patients with different histories of T1D in terms of duration and type of insulin treatment (insulin pump *versus* multiple daily injections). Despite knowing Eversense XL^®^ meters are not routinely used during pregnancy, we intend to report that CGM through a long-term subcutaneous implantable sensor in patients with T1D resulted in satisfactory achievement of composite glycaemic outcomes during the 2nd and 3rd trimesters of pregnancy, apart from a longer time spent below the glycaemic target than recommended by the International Guidelines [6]. However, this did not entail an increased risk for the course of pregnancy, the delivery process, or the health of the newborn. No problems were elicited after the removal and reimplantation process of the subcutaneous sensor in the pregnant patients, nor in the fetuses.

In conclusion, this is the first report in the literature of Eversense XL^®^ use for glucose monitoring during pregnancy, expanding the range of CGM options suitable for women with T1D and offering reassuring real-life clinical data about both maternal and fetal outcomes; however, further research is warranted to confirm safety and efficacy in larger populations.

## AUTHORS’ CONTRIBUTIONS

The authors confirm their contributions to the paper as follows: Study conception and design: B.A.C and B.N.; data collection: B.N., R.V., G.A., M.F., C.A., V.W. and O.E.; analysis and interpretation of results: B.N, B.A.C., and R.V.; draft manuscript: B.N. and B.A.C.

All authors reviewed the results and approved the final version of the manuscript.

## Figures and Tables

**Table 1:** Blood and anthropometric parameters, sensor data, and obstetrician parameters throughout a 33-year-old type 1 diabetic patient’s first pregnancy.

**Gestational Period**	**Blood and Anthropometric Parameters**		**Sensor Data**					**Foetal Growth ** **Parameters**		
	**Body Weight, kg**	**HbA1c^#^, mmol/mol (%)**	TIR (%)*	**TAR (%)°**	**TBR (%)^§^**	**Mean glycaemia, mg/dL**	**Standard Deviation**	**AFI^+^, cm**	**AC^ç^, mm**	**Estimated Weight, Grams (percentile)**
**Pregestational (23/08/2022)**	54.0	**56 (7.3)**	**65.8**	26.3	7.9	152	56.0	-	-	-
**I trim.** **(15/11/2022)**	55.5 (+1,5)	48 (6.7)	72.0	**22.2**	**5.8**	126	41.2	-	98.5	136 (16.9°)
**II trim.** **(09/02/2023)**	65.0 (+11,0)	42 (6.0)	78.6	16.8	**4.6**	123	45.5	13.5	220.4	1052 (37.3°)
**III trim.** **(28/04/2023)**	69.0 (+15,0)	42 (6.0)	76.5	19.7	3.8	115	36.0	10.0	290	2546 (39.7°)
**Postpartum** **(25/05/2023)**	63.0	44 (6.2)	74.3	18.4	**7.3**	128	47.6	-	-	-

**Note:** **^#^ HbA1c** = glycated haemoglobin * **TIR** = Time In Range: 63-140 mg/dL in Pregnancy, 70-170 mg/dL before and after Pregnancy (target >70%) ° **TAR** = Time Above Range: >140 mg/dL in Pregnancy, >170 mg/dL before and after Pregnancy (target <25%) ^§^**TBR** = Time Below Range: <63 mg/dL in Pregnancy <70 mg/dL before and after Pregnancy (target <4%) **^+^ AFI** = Amniotic Fluid Index **^ç^ AC** = Abdominal Circumference.

**Table 2:** Blood and anthropometric parameters, sensor data, and obstetrician parameters throughout a 37-year-old type 1 diabetic patient’s second pregnancy.

**Gestational Period**	**Blood and Anthropometric Parameters**		**Sensor Data**					**Foetal Growth ** **Parameters**		
	**Body Weight, kg**	**HbA1c^#^, mmol/mol (%)**	TIR (%)*	**TAR (%)°**	**TBR (%)^§^**	**Mean Glycaemia, mg/dL**	**Standard Deviation**	**AFI^+^, cm**	**AC^ç^, mm**	**Estimated Weight, Grams (percentile)**
**Pregestational (23/05/2023)**	54.5	46 (6.4)	75.1	23.7	1.2	126	41.4	-	-	-
**I trim.** **(31/08/2023)**	57.5 (+3)	43 (6.1)	72.0	**26.1**	1.9	121	46.2	-	104.2	(38.0°)
**II trim.** **(16/11/2023)**	63.4 (+8,9)	32 (5.1)	79.4	16.4	**4.2**	106	33.5	11.5	225.2	1005 (36.1°)
**III trim.** **(25/01/2024)**	68.6 (+14,1)	36 (5.4)	77.5	16.1	**6.4**	107	38.9	14.4	307.0	2422 (30.8°)
**Postpartum** **(9/04/2024)**	61.0	42 (6.0)	81.8	5.0	4.2	118	29.5	-	-	-

**Note:** **^#^ HbA1c** = glycated haemoglobin * **TIR** = Time In Range: 63-140 mg/dL in Pregnancy, 70-170 mg/dL before and after Pregnancy (target >70%) ° **TAR** = Time Above Range: >140 mg/dL in Pregnancy, >170 mg/dL before and after Pregnancy (target <25%) ^§^**TBR** = Time Below Range: <63 mg/dL in Pregnancy <70 mg/dL before and after Pregnancy (target <4%) **^+^ AFI** = Amniotic Fluid Index **^ç^ AC** = Abdominal Circumference.

## Data Availability

The data and supportive information are available within the article.

## LIST OF ABBREVIATIONS

AC: Abdominal Circumference
AFI: Amniotic Fluid Index
BMI: Body Mass Index
CSII: Continuous Subcutaneous Insulin Infusion
HbA1c: Glycated Haemoglobin
HCL: Hybrid Closed Loop
LGA: Large for Gestational Age
MARD: Mean Absolute Relative Difference
MDI: Multiple Daily Injections
NICE: National Institute for Health and Care Excellence
rt-CGM: Real-Time Continuous Glucose Monitoring
T1D: Type 1 Diabetes Mellitus
TAR: Time Above Range
TBR: Time Below Range
TIR: Time In Range
TSH: Thyroid-Stimulating Hormone

## STANDARDS OF REPORTING

CARE guidelines were followed.
